# A Mixed-Methods Evaluation of Usability and Acceptability of Web-Based Relapse Prevention and Self-Monitoring Program: Secondary Analysis of a Pilot Randomized Controlled Trial

**DOI:** 10.3389/fpsyt.2021.625480

**Published:** 2021-02-11

**Authors:** Ayumi Takano, Toshihiko Matsumoto

**Affiliations:** ^1^Department of Mental Health and Psychiatric Nursing, Tokyo Medical and Dental University, Tokyo, Japan; ^2^Department of Drug Dependence Research, National Center of Neurology and Psychiatry, Tokyo, Japan

**Keywords:** web-based intervention, drug dependence, relapse prevention, self-monitoring, usability, acceptability

## Abstract

This secondary analysis of a pilot randomized controlled trial assessed the usability and acceptability of a web-based relapse prevention and self-monitoring program, which was newly developed for Japanese drug users. We analyzed quantitative and qualitative data using a mixed-method approach among 43 study participants. We assessed the usability of the programs using the Web Usability Scale (WUS) and satisfaction and acceptance using the Client Satisfaction Questionnaire 8-item version (CSQ-8). We also collected qualitative feedback using semi-structured open-ended questions. The results of WUS indicated that the usability of the programs was sufficient. According to the CSQ-8 results, the self-monitoring program had less satisfaction, while the web-based relapse prevention program met satisfaction. Some possible improvements for a future version of the web-based programs were suggested based on participants' feedback.

## Introduction

Despite evidence supporting psychosocial treatment for drug abuse and dependence, there is still a treatment gap between potential treatment needs and actually provided treatment (1). To deal with challenges in treatment implementation, e-health interventions, which are therapeutic interventions using information and communication technologies (ICT), have been adapted to drug use problems (2). Various types of e-health interventions, such as computer-assisted, web-, or mobile-based programs for drug users, have been developed based on psychosocial approaches as well as for use in face-to-face treatment. These e-health programs have demonstrated benefit for abstinence, treatment retention, and cost-effectiveness with small to moderate effect sizes (2–4). However, the majority of these programs targeted opioid, cocaine, or cannabis users in western countries (5–11). There have been few e-health programs in Asian countries, where the national drug policy is likely to be very strict, and there are obstacles to seeking treatment because of a strong stigma.

The authors developed a web-based treatment program named “e-learning Serigaya Methamphetamine Relapse Prevention Program (e-SMARPP)” for Japanese drug users (12). The content of e-SMARPP was based on an existing face-to-face relapse prevention program, the Serigaya Methamphetamine Relapse Prevention Program (SMARPP), which is in widespread use in Japan. The web-based program was developed to be user friendly, includes a user guide, support information, FAQ, and contact form. The website was designed to support all devices (PC, mobile phone, tablet). We tested the effectiveness of e-SMARPP with a pilot randomized controlled trial (RCT) (13, 14). We found a medium effect on abstinence, although the statistically-significant effect was not confirmed because of the small sample size (14). In the RCT, the intervention group was asked to complete a web-based 6-session relapse prevention program with 8-week self-monitoring. The control group was only asked to complete a web-based 8-week self-monitoring program. The relapse prevention program involved interactive therapist support. The completion rates of the intervention were high; relapse prevention program: 73.9%, self-monitoring: 82.6% in the intervention group, 100.0% in the control group (14). However, the usability and acceptability of e-SMARPP have not been evaluated in detail.

The aim of this secondary analysis of the pilot RCT was to assess the usability and acceptability of the web-based relapse prevention and self-monitoring program, which was newly developed for Japanese drug users. We analyzed quantitative and qualitative data using a mixed-method design to obtain information to improve future implementation.

## Methods

### Trial Design

This study analyzed the usability and satisfaction of a previous RCT, which evaluated the effectiveness of a web-based treatment program (14). The design was a two-arm (one-to-one allocation ratio), parallel-group, non-blinded, and multicenter randomized controlled trial. Further methodology and results detailing the efficacy and completion rate of the original RCT can be found in our published articles (13, 14). The Ethics Committee of the Faculty of Medicine and Graduate School of Medicine of the University of Tokyo and the Institutional Review Boards of each recruiting hospital and clinic approved this study. The study protocol was registered with the University Hospital Medical Information Network clinical trial registry (UMIN000016075).

### Participants

Participants were recruited from six psychiatric hospitals located in large cities of Japan, with all hospitals providing specialized treatment for those with substance use disorders. Outpatients were required to be diagnosed with substance use disorder by DSM-IV/DSM-5 in addition to having used any type of illicit drug in the past year to be eligible for this study. Participants also had to be able to use the internet in order to access the e-SMARPP website and communicate via email. Exclusion criteria were patients with severe physical diseases, high suicide risk, severe substance-induced psychotic disorder symptoms, or impaired cognitive function. Eligible participants were then randomly allocated to either the e-SMARPP intervention group or the control group utilizing the permuted block method. The researchers and staff working at the psychiatric hospitals were blinded to the results of the randomization.

### Intervention

The main intervention modules of e-SMARPP were the cognitive behavioral relapse prevention sessions, and web-based self-monitoring practice. The relapse prevention sessions included six sessions, and each had three videos and homework assignments. Participants were required to submit two homework assignments and a diary after each assignment via the e-SMARPP website. Participants would then receive tailored feedback comments from healthcare professionals trained to support patients with substance use disorder to enhance user motivation and provide individual support. Automated notification emails were also sent to the participant when submitting homework or receiving feedback. The web-based self-monitoring was a self-guided module in which the participants recorded their daily drug use on a calendar format for 8 weeks.

Participants assigned to the intervention group received the complete content of the e-SMARPP intervention. Although participants could progress at their own pace, all sessions were expected to be completed within an 8-week intervention period. A daily record of drug use was also kept. An email reminder was sent to the participant by the web-therapist if they missed a weekly deadline. Those in the control group only participated in the self-monitoring module.

### Measures and Statistical Analysis

Demographic characteristics included age, sex, education level, employment status, and internet use (frequency and device). Clinical characteristics included primary abused drug, total abstinent days from all substances in the past 28 days, and drug dependence severity assessed using Drug Abuse Screening Test-20 (15, 16).

After the intervention, the participants were asked to complete a feedback survey. Participants evaluated the usability and satisfaction of the program using the following measurements. The e-SMARPP website was evaluated using the Web Usability Scale (WUS) (17). WUS is comprised of 21 items measured on a 5-point scale (1: disagree to 5: agree) and seven subscales: ease of use, website structure, visual, response speed, favorability, helpfulness, and credibility. Subscale average scores were also calculated, with higher scores indicating higher website usability. The Client Satisfaction Questionnaire 8-item version (CSQ-8) was used to assess perceived program satisfaction and acceptance (18). CSQ-8 consists of 8 items measured on a 4-point scale. A higher score indicates higher satisfaction with service use. Demographic and clinical characteristics, WUS, and CSQ-8 were compared by the intervention condition using a *t*-test (the relapse prevention program vs. self-monitoring).

For an in-depth evaluation of usability and acceptability, qualitative feedback was also collected using the following semi-structured open-ended questions: general impressions of the program, the settings in which they had used the program, technical difficulties, program features they felt were the most and least useful, and suggestions for future versions of the program. We summarized the positive feedback comments by program content or function and categorized suggestions for improvements using a content analysis approach. The initial codes were extracted from text data of feedback comments. Related codes were merged together into sub-categories and main categories according to similarities. When analyzing the feedback comments, we combined the comments from both groups because this facilitated the collection of major points about how to revise the program, rather than determining the differences in responses between groups.

## Results

### Participant Demographics

The participants' mean age was 38.3 (SD: 7.4), and 68.5% were male. Approximately 54% were unemployed. Among the participants, 83.3% used the internet every day, and 73.2% accessed the internet using their smartphone. About half of the participants mainly used methamphetamine. There was no significant difference in the characteristics between the two groups. The participants' demographic characteristics by group are provided in the Supplementary Table 1.

### Usability and Satisfaction

In total, 43 participants completed the feedback survey. Among the participants, 83.7% (*n* = 36) accessed e-SMARPP from their home, and 9.3% (*n* = 4) used it when away from home. To access e-SMARPP, 48.8% (*n* = 21) used their smartphone and 44.2% (*n* = 19) used a PC.

**Figure 1:**
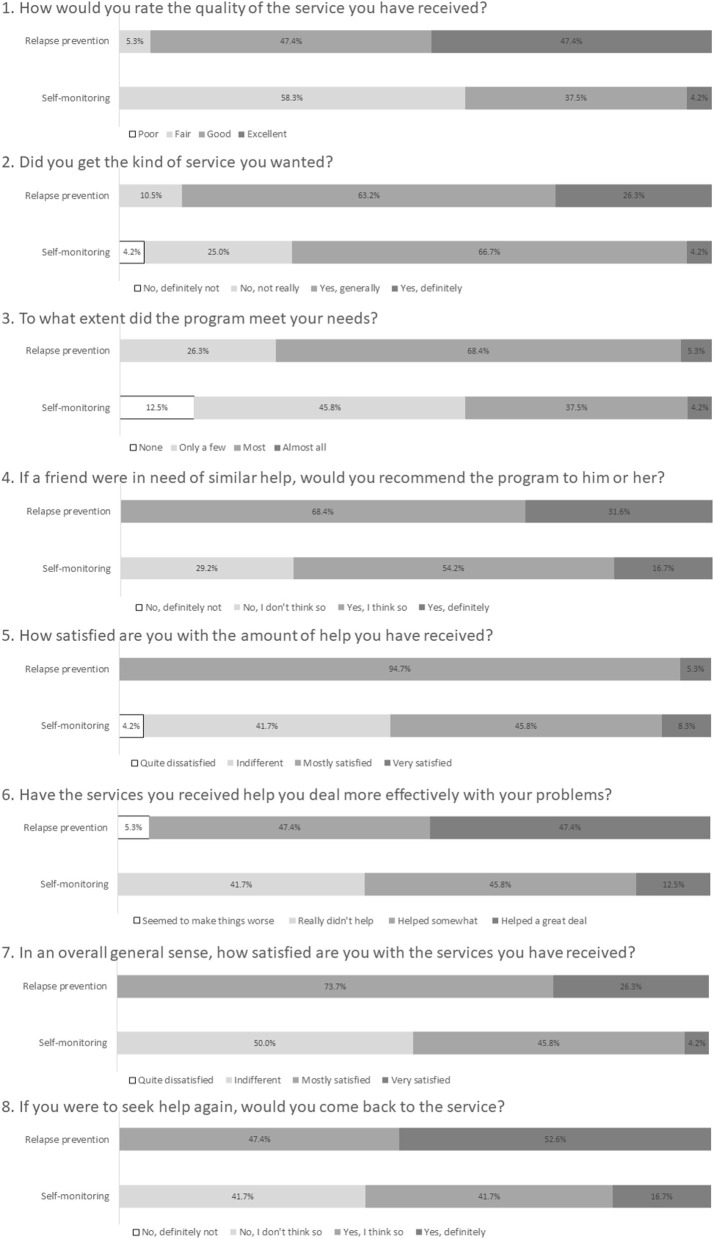
Detailed program satisfaction assessed using CSQ-8.

Table 1 shows the results of the usability and satisfaction scores of e-SMARPP by the intervention condition. All scores of the WUS subscales were over three points for program usability, except for the favorability subscale in the self-monitoring group. In the relapse prevention group, the subscales of ease of use, visual, and credibility were over four points. In the self-monitoring group, only the response speed sub-scale was over four points. The subscales of favorability and credibility of the relapse prevention group were significantly higher than those of the self-monitoring group. As for program satisfaction, the total score of CSQ-8 in the relapse prevention group was significantly higher than the self-monitoring group. Figure 1 shows detailed responses for each item in CSQ-8. All participants assigned to the relapse prevention program felt the program was “mostly satisfied” or “very satisfied.” They said they would come back to the program if they seek treatment again. Moreover, they said they would recommend it to their friends who need similar help. On the other hand, about half of the participants assigned to the self-monitoring program felt the program did not meet their needs and were less satisfied.

**Table 1:** Program usability and satisfaction by intervention condition.

		**Relapse prevention (*****n*** **=** **19)**		**Self-monitoring (*****n*** **=** **24)**		
		**Mean**	**SD**	**Mean**	**SD**	***p***
WUS, total score		3.9	0.3	3.7	0.6	0.08
Subscale						
Ease of use		4.0	0.7	3.9	0.9	0.71
Website structure		3.7	0.9	3.7	0.9	0.98
Visual		4.0	0.8	3.6	0.8	0.14
Response speed		3.8	1.0	4.2	0.9	0.14
Favorability		3.7	0.6	2.9	1.0	0.002
Helpfulness		3.9	0.5	3.8	0.6	0.41
Credibility		4.4	0.5	3.6	0.8	0.001
CSQ-8, total score		25.9	2.5	21.0	4.1	<0.001
Item						
#1	How would you rate the quality of the service you have received?	3.4	0.6	2.5	0.6	<0.001
#2	Did you get the kind of service you wanted?	3.2	0.6	2.7	0.6	0.022
#3	To what extent did the program meet your needs?	2.8	0.5	2.3	0.8	0.033
#4	If a friend were in need of similar help, would you recommend the program to him or her?	3.3	0.5	2.9	0.7	0.021
#5	How satisfied are you with the amount of help you have received?	3.1	0.2	2.6	0.7	0.009
#6	Have the services you received help you deal more effectively with your problems?	3.4	0.8	2.7	0.7	0.005
#7	In an overall general sense, how satisfied are you with the services you have received?	3.3	0.5	2.5	0.6	<0.001
#8	If you were to seek help again, would you come back to the service?	3.5	0.5	2.8	0.7	<0.001

*WUS: web usability scale. A higher score indicates higher usability assessed on a 5-point Likert scale (1: disagree to 5: agree)*.

*CSQ-8: client satisfaction questionnaire 8-item version. A higher score indicates higher satisfaction assessed on a 4-point Likert scale (1: less satisfied to 4: more satisfied)*.

### Qualitative Feedback

The results of the qualitative feedback are shown in Tables 2, 3. Overall, the participants provided positive feedback comments, especially for therapist involvement and relapse prevention program content. Meanwhile, potential challenges and areas for improvement were revealed. Some participants stated that a smartphone App would be more useful and interesting. Non-active drug users stated the self-monitoring program was not helpful because they had already quit drugs for several months and did not feel drug cravings. We received some suggestions for a future version of e-SMARPP. As for self-monitoring, some participants suggested that structured self-monitoring for triggers would be of great help. Additional content and functions were suggested, such as relaxation exercises, information for LGBTQ, or peers' narrative stories. As for interaction, some participants proposed online sessions with therapists or peers. A portion of participants felt the 2-month program duration was short, and that a longer duration (e.g., 6 months) would be better.

**Table 2:** Positive feedback comments for the web-based program.

**Content**	**Comment examples**
Overall	• I was able to use the program when I was alone.
	• The program was easy to access from anywhere.
	• Even if I am unable to go to a therapy session physically, I could sit in front of the computer and slowly face myself little by little.
	• I've felt lighthearted more often since starting the program.
**Relapse prevention**	
Therapist involvement	• I was happy that there was thoughtful feedback after each homework assignment.
	• The feedback was provided very quickly.
	• The feedback for my homework was suitable and precise.
	• The balance between internet tasks and human interaction was just right.
	• I felt that I was not alone.
	• The emotional distance between the therapist and me felt very good.
	• After reading the feedback, at times, I felt confident, and at other times I felt I had to keep working hard. It was very encouraging.
	• Sometimes the advice I received helped me to calm my negative emotions.
Contents:videos, homework	• The program helped me to prepare for face-to-face therapy and was a good opportunity for me to re-evaluate the problems I am dealing with.
	• The videos were very thorough and rich in content.
	• The combination of videos and assignments allowed me to think step-by-step about how to deal with drugs.
	• I was able to organize the emotions inside of myself while doing the homework.
	• I felt that I was less likely to be tempted by my weakness because I was spending more time reflecting and thinking about drugs.
	• There were many things that were very applicable to myself, such as the thought patterns of those with addictions.
Self-monitoring	• Being able to use the calendar with one click was convenient.
	• When I went from a period of continued drug use where the screen was full of red, to a period of reduced drug use and a completely blue screen, I felt that I was making progress, even if it was just a small step^*^.

* *Days of primary drug use were marked as red, other drugs/alcohol use was marked as yellow, and no drug/alcohol use was marked as blue*.

**Table 3:** Suggestions for a future version.

**Category**	**Sub-category**	**Comment examples**
Application feature	• Mobile-based application • Familiar interface • Entertainment elements	• It would be easier to use in an app format on a smartphone.
		• Make it easier to type on a smartphone. If you could type like you can on Twitter, it may be less stressful.
		• It would be nice if it could be used like a game app, as part of my life or for entertainment.
More useful self-monitoring	• Useful self-monitoring for people maintaining abstinence	• Perhaps because I've been clean for a relatively long time now and I don't currently feel much of a desire to use drugs, I didn't feel a strong sense of accomplishment when using the self-monitoring feature.
	• Easy to use free-writing section	• If I had to leave a comment for each daily record, I might have been able to maintain my motivation.
		• We were free to write anything in the comments section, but it may have been easier if there were some example formats, such as about triggers.
Additional contents	• Information about recovery and drug policy	• It would be nice to have access to some simple information about recovery. For example, a quick look at the theme of the day to be used in NA, etc.
		• Information about changes in drug laws, or stories of reformed substance users overcoming their addiction would be helpful.
	• Messges of encouragement	• Even if it is just a proverb, it would be nice if there was a SMARPP theme of the week message or something similar, without being too obtrusive.
	• Meditation content	• I think meditation is quite useful, and may have used the program more if there were videos on meditation for beginners or simple exercise/breathing videos.
	• Optional content for LGBTQ	• Add a section on drugs related to sexual minorities, and make it available to those who are applicable or wish to use it.
	• Video refinement	• The videos should be reenactments or dramatic representations by Japanese people.
		• I think it would be easier to answer questionnaires and assignments more deeply if there were pictures of the staff and teachers, as I would feel a sense of affinity.
	• Point system for abstinence	• It may be interesting if there was a system such as receiving tips for each day I was clean like in AA, as I would be able to challenge myself.
Online interaction	• Online session or communication with therapists or peers	• I thought that if therapists and participants could have Skype sessions or something similar, it would be good training for gradually getting out and forming relationships.
		• Counseling for people who can't finish their assignments.
		• I would have liked to be able to have some form of communication with other partipicants. I think that because it's online, it would be easier to share our opinions and let our feelings out without worrying.
Program duration		• I thought that 6 sessions in total was short. • Especially because it can be done from home, I wanted to take more time (at least 6 months). Extend the period of use.

## Discussion

This study evaluated the usability and acceptability of a web-based relapse prevention and self-monitoring program among 43 Japanese drug users who participated in a pilot RCT. The usability of both programs was good. The majority of the participants felt these programs were easy to use, helpful, and credible. The participants who used the relapse prevention program were satisfied with the content. However, participants who only used the self-monitoring program were likely to be unsatisfied. The participants provided helpful suggestions for a future version of e-SMARPP.

### Usability

WUS results indicated that both web-based relapse prevention and self-monitoring programs were user-friendly. The interface of e-SMARPP was simple and it was easy to find desired content. In the self-monitoring calendar, the daily record was displayed in three colors (red: primary drug use, yellow: other drugs/alcohol use, blue: no drug/alcohol use). Through this display, participants could see at a glance their drug use patterns. Moreover, the participants quickly became familiar with actions on the website because automated notification emails were sent when submitting homework or receiving feedback. These functions might help to increase participant use of the programs. However, some participants felt it would be easier to use the program through a smartphone app, as apps are now increasingly common. In Japan, over 95% of people aged 20–49 use the internet, and about 80% use their smartphone when accessing the internet (19). Globally, smartphone-based applications have been developed as educational tools, self-monitoring for substance use and craving, screening and brief intervention, harm reduction strategy for people with at-risk for addictive behavior, support for recovery from addiction issues, or linkage to peers in self-help group (20–22). A smartphone app might be better if we develop an internet-based intervention with frequent use or a long intervention duration, such as daily self-monitoring. Most existing smartphone-based apps for addiction problems are for alcohol problems and few are for drug problems, and the efficacy of apps for drug problems is unclear (21, 23). Attractive features might be needed because mobile-based interventions are used less and less as time passes unless regular contact and prompts are provided (23).

### Satisfaction and Acceptability

The results of the CSQ-8 and qualitative feedback suggested that participants were satisfied with the web-based relapse prevention program, and especially interactions with the therapist and program content. Previous studies revealed that therapist involvement in computer-delivered intervention improved treatment outcomes and retention (24–27). The interaction with the therapist might enhance the participants' motivation to continue treatment and to improve satisfaction. It might be a good idea to include additional interactive functions such as an online peer meeting or counseling according to the intervention purpose. There might still be room for improvement in the web-based self-monitoring program. The participants might have at times felt that the program was less helpful and insufficient to motivate behavioral change because the web-based self-monitoring program was too simple and did not include interaction with the therapist. Additional useful content (e.g., self-monitoring of drug craving and triggers, text boxes for free comments) or regular automated prompts might be needed if the web-based self-monitoring program does not include interactions with others, especially for drug users who have maintained abstinence for several months.

### Limitations

There are some possible limitations to this study. First, the sample size in this study was small, and the participants were recruited in limited areas of Japan. These limitations could lead to uncertainty in the representativeness and the generalization of the results. Second, satisfaction with the web-based self-monitoring program might be affected by the primary abused drug because the participants used different drugs with different frequencies. Further study with a large sample is needed to assess the usability and acceptability of the web-based program.

## Data Availability Statement

The datasets presented in this article are not readily available because we cannot share the data unless the ethics committee approves data sharing. Requests to access the datasets should be directed to Ayumi Takano, ayumi-takano@umin.ac.jp.

## Ethics Statement

The studies involving human participants were reviewed and approved by The Ethics Committee of the Faculty of Medicine and Graduate School of Medicine of the University of Tokyo. The patients/participants provided their written informed consent to participate in this study.

## Author Contributions

AT contributed to data collection and analysis and drafted the manuscript. TM approved the use of the existing face-to-face program and advised on development of the web-based program. AT and TM contributed to interpretation and critically reviewed the manuscript. All authors approved the final version of the manuscript.

## Conflict of Interest

The authors declare that the research was conducted in the absence of any commercial or financial relationships that could be construed as a potential conflict of interest.
