# Supplementary material for: A Mixed-Methods Evaluation of Usability and Acceptability of Web-Based Relapse Prevention and Self-Monitoring Program: Secondary Analysis of a Pilot Randomized Controlled Trial
Source: Front Psychiatry. 2021 Feb 11;12:625480. doi: 10.3389/fpsyt.2021.625480 (PMC7905227; doi:10.3389/fpsyt.2021.625480)
Supplement: Supplementary file 1 [file Table_1.DOCX]

Supplementary table

Participants’ demographic characteristics at the baseline

|  |  | Relapse prevention (n=23) | | Self-monitoring (n=25) | |
| --- | --- | --- | --- | --- | --- |
|  |  | n/mean | %/SD | n/mean | %/SD |
| Age (mean and SD) |  | 37.0 | 7.3 | 39.5 | 7.5 |
| Sex | Male | 14 | 60.9% | 19 | 76.0% |
| Marital status | Currently married | 4 | 17.4% | 5 | 20.0% |
|  | Never married | 15 | 65.2% | 17 | 68.0% |
|  | Divorced | 4 | 17.4% | 3 | 12.0% |
| Cohabiter | Single | 4 | 17.4% | 7 | 28.0% |
| Education | Middle school | 2 | 8.7% | 4 | 16.0% |
|  | High school | 9 | 39.1% | 4 | 16.0% |
|  | Some college | 6 | 26.1% | 7 | 28.0% |
|  | College or higher | 6 | 26.1% | 10 | 40.0% |
| Employment status | Full-time | 4 | 17.4% | 3 | 14.6% |
|  | Part-time | 5 | 21.7% | 2 | 8.0% |
|  | Unemployed | 12 | 52.2% | 14 | 56.0% |
|  | Sick leave | 0 | 0% | 2 | 8.0% |
|  | Housewife/other | 2 | 8.7% | 4 | 16.0% |
| Internet use | Every day | 19 | 82.6% | 21 | 84.0% |
|  | 2 hours or more/day | 15 | 65.2% | 18 | 72.0% |
| Internet device  (most use) | Smartphone | 18 | 78.3% | 17 | 68.0% |
|  | Personal computer | 4 | 17.4% | 7 | 28.0% |
|  | Tablet/mobile phone | 1 | 4.3% | 1 | 4.0% |
| Primary abused drug | Methamphetamine | 13 | 56.5% | 11 | 44.0% |
|  | NPS | 1 | 4.3% | 5 | 20.0% |
|  | MDMA | 3 | 13.0% | 2 | 8.0% |
|  | Hypnotics/anxiolytics | 1 | 4.3% | 3 | 12.0% |
|  | Cough medicine | 2 | 8.7% | 2 | 8.0% |
|  | Heroine | 0 | 0% | 2 | 8.0% |
|  | Inhalant | 1 | 4.3% | 0 | 0% |
|  | Poly drug | 2 | 8.7% | 0 | 0% |
| Total abstinent days from all substances in the past 28 days (mean and SD) | | 25.2 | 7.0 | 23.0 | 9.3 |
| Drug dependence severity (DAST-20) | Total score (mean and SD) | 13.2 | 3.6 | 11.7 | 3.9 |
|  | Low (1-5) | 1 | 4.3% | 3 | 12.0% |
|  | Intermediate (6-10) | 4 | 17.4% | 4 | 16.0% |
|  | Substantial (11-15) | 14 | 60.9% | 13 | 52.0% |
|  | Severe (16-20) | 4 | 17.4% | 5 | 20.0% |

NPS: New psychoactive substances

MDMA: 3,4-methylenedioxymethamphetamine

DAST: Drug Abuse Screening Test
